# The Regulation of Seventeen Inflammatory Mediators are Associated with Patient Outcomes in Severe Fever with Thrombocytopenia Syndrome

**DOI:** 10.1038/s41598-017-18616-z

**Published:** 2018-01-09

**Authors:** Li-Fen Hu, Ting Wu, Bo Wang, Yuan-Yuan Wei, Qin-Xiang Kong, Ying Ye, Hua-Fa Yin, Jia-Bin Li

**Affiliations:** 1 Department of Infectious Diseases, the First Affiliated Hospital of Anhui Medical University, Hefei, Anhui China; 2 Department of Infectious Diseases, the Chaohu Affiliated Hospital of Anhui Medical University, Chaohu, Anhui China

## Abstract

Severe fever with thrombocytopenia syndrome (SFTS) as an emerging infection disease results in high morbidity and mortality in China. In this study, the circulating levels of 36 inflammatory mediators in 33 SFTS patients on days 3–7, 8–12 and 13–20 post-illness were measured by a multiplex Luminex® system dynamically. Among the patients, 15 severe patients recovered, 11 severe patients died within three weeks. We found IL-1RA, IL-6, IL-15, IL-10, TNF-α, IFN-γ, G-CSF, eotaxin, IL-8, IP-10, MCP-1, MIP-1α, MIP-1β and fractalkine were significantly upregulated in SFTS patients. Elevated IL-15 and eotaxin in SFTS patients were reported firstly. The highest levels of pro-inflammatory and anti-inflammatory cytokines coexisted in fatal patients during the first week. Inflammatory mediators remained high levels when death occurred in fatal patients, they were recovered within three weeks in nonfatal patients. Our results showed the occurrence of inflammatory storm in SFTS patients were associated with the severity of SFTS. RANTES and PDGF were down regulated and remained significantly lower levels in fatal patients throughout the course of disease, the concentrations of RANTES and PDGF were remarkably positively correlated with the platelet count. Our results demonstrated that dysregulated inflammatory response was associated with disease pathogenesis and mortality in SFTS patients.

## Introduction

Severe fever with thrombocytopenia syndrome (SFTS) is an emerging infectious disease caused by a novel bunyavirus which is referred to as SFTS virus (SFTSV)^1^. The disease was first identified in 2009 in rural areas of central China, tick sting appears to be a major risk factor for acquiring SFTS^2^. The disease has now been epidemic in 20 provinces in China and also been observed in Japan and Korea^1–4^. SFTS is an acute-onset disease in most patients with major clinical symptoms, including acute fever, thrombocytopenia, leukocytopenia, gastrointestinal symptoms, neural disorders, elevated lactate dehydrogenase (LDH), and creatine kinase (CK), the illness can quickly progress to multiple organ dysfunction syndrome (MODS), death usually occurs within 2 weeks of the onset of SFTS^5^. Case-fatality rates were varied between 2.5% and 33.3% in China, while mortality rates of 36.3% in Korea and 41.5% in Japan have been reported^6–9^.

SFTS is now an expanding public health threat in China due to its increased incidence area and high mortality. Presently, the pathogenesis of SFTS with rapid disease progression is not yet clear. Immune response against viral infection is believed to play an important role in its pathogenesis^10–12^. Previous studies have indicated that cytokines including IL-1RA, IL-6, IL-10, G-CSF, IP-10, TNF-α, IFN-γ, and MCP-1 were elevated in SFTS patients^13–16^.

However, the dynamic changes of inflammatory factors and virus load with the disease progression in SFTS patients are less clear, which is important for clinicians to evaluate the disease prognosis. To understand the contribution of the inflammatory response to disease severity and seek experimental evidence to guide future therapeutic strategies, we continuously measured the production of 36 important inflammatory mediators and the virus load in blood samples collected from SFTS patients with different prognoses.

## Methods

### Patients and Clinical Samples

This prospective study included thirty-three patients in the Infectious Disease Department of the First Affiliated Hospital of Anhui Medical University who were diagnosed with SFTS with SFTSV RNA-positive from March 2015 to July 2016. Informed consent was obtained from all patients, in accordance with the Declaration of Helsinki. The study was approved by the local Ethics Committee of Anhui Medical University, and all experiments in this research were performed in accordance with the relevant guidelines and regulations. Informed consent for a single blood sample was also obtained from healthy (control) volunteers. According to the guidelines for the prevention and treatment of fever with thrombocytopenia syndrome (2010 Edition)^17^, patients were diagnosed as having severe or mild form of disease. In severe patients, survivors were categorized as the nonfatal severe group, while those who died were classified as the fatal group. Throughout the disease course, at 3–7 days, 8–12 days and 13–20 days post-disease onset, blood samples were collected unless the patient died or was discharged after recovery. Blood samples from 10 healthy volunteers were also analysed as control samples. The mean age of the healthy controls was 60.5 ± 10 years, and 6 (60%) were male, there was no significant difference in age and gender between healthy controls and SFTS patients.

### Quantitative Real-time PCR

RNA was extracted from plasma using a high-purity viral RNA kit (Qiagen) according to the manufacturer’s instructions. SFTS virus was amplified using specific primers and probes by real-time reverse-transcription polymerase chain reaction (RT-PCR) under conditions previously described^18^.

### Measurement of Inflammatory Mediators

Inflammatory mediators were measured in plasma samples of patients and controls using MILLIPLEX® MAP human cytokine/chemokine magnetic bead panel kits (Merck Millipore, Germany) according to the manufacturer’s instructions (Luminex® 200™ System, Life Technologies, Grand Island, NY). The following inflammatory mediators were measured: interleukin (IL)-1β, IL-1α, IL-1 receptor antagonist (IL-1RA), IL-2, IL-3, IL-4, IL-5, IL-6, IL-7, IL-9, IL-10, IL-12 (p40), IL-12 (p70), IL-13, IL-15, IL-17A, human soluble CD40 ligand (sCD40L), FMS such as tyrosine kinase 3 ligand (Flt-3L), tumour necrosis factor (TNF)-α, TNF-β, transforming growth factor-α (TGF-α), granulocyte colony-stimulating factor (G-CSF), granulocyte macrophage colony-stimulating factor (GM-CSF), interferon (IFN)-γ, platelet-derived growth factor (PDGF-AA), PDGF-AB/BB, eotaxin, IL-8, IFN-γ-inducible protein (IP-10), monocyte chemotactic protein-1 (MCP-1), MCP-3, macrophage inflammation protein 1a (MIP-1α), macrophage inflammation protein-1β (MIP-1β), regulated on activation and normally T-cell expressed (RANTES), fractalkine and macrophage-derived chemokine/CCL22 (MDC*)*.

### Statistical Analyses

Between the 3 groups of mild, nonfatal severe and fatal patients during 3 time periods, comparisons of clinical parameters and levels of inflammatory mediators were calculated by unpaired t test, non-parametric tests (for continuous variables) or Pearson’s χ2 test on a cross table (for categorical variables), where appropriate, using GraphPad Prism 5.0 (GraphPad software, San Diego, USA). Logistic regression analysis was used to show correlations between platelet count and certain cytokines. A two-sided *P*-value < 0.05 was considered statistically significant.

## Results

### Characterization of SFTS Patients

There were no deaths among the 7 mild patients, while 11 of the 26 severe patients died. In the first week post-illness, 1 patients died with complications of pulmonary infection, acute pancreatitis and MODS, 1 patients died with complications of hemorrhage of digestive tract and MODS; on day 8–12, 2 patients died with complications of pulmonary infection and 2 patients died with complications of MODS; on days 13–20 of disease, 2 patients died with complications of MODS and diffuse intravascular coagulation (DIC), 2 patients died with complications of MODS and pulmonary infection. One patient died on day 21 post-illness.

As summarized in Table 1, patients were between 17 to 86 years old; the average age of the fatal patients was significantly higher than that of the nonfatal patients, fatal patients have higher incidences of conscious disorder, hemorrhage of digestive tract, pulmonary infection, acute pancreatitis and DIC, however, higher incidences of vomiting and diarrhoea in nonfatal severe patients were noted.

**Table 1:** Comparison of clinical characteristics between fatal and nonfatal patients of severe fever with thrombocytopenia syndrome.

Index*	Death (N = 11)	Nonfatal severity (N = 15)	Mildness (N = 7)	*p* ^a^	*p* ^b^	*p* ^c^
Age, years	69.36 ± 10.07	56.2 ± 14.8	53 ± 18.9	0.013	0.028	0.696
Male, N (%)	54.5	40	57.1	0.368	0.417	0.181
Fever, N (%)	100	100	100	—	—	—
Headache, N (%)	27.3	40	14.3	0.402	0.485	0.243
Vomit, N (%)	36.4	73.3	28.6	0.069	0.572	0.064
Diarrhea, N (%)	36.4	53.3	28.6	0.324	0.572	0.268
Conscious Disturbance, N (%)	100	26.7	0	0	0	0.187
Hemorrhage of digestive tract, N (%)	36.4	20	14.3	0.313	0.324	0.622
Pulmonary infection, N (%)	45.5	33.3	14.3	0.412	0.199	0.349
MODS, N (%)	63.6	20	0	0.032	0.01	0.295
Acute pancreatitis, N (%)	36.4	26.7	28.6	0.457	0.572	0.651
Oral candidiasis, N (%)	0	13.3	0	0.323	—	0.455
DIC, N (%)	18.2	0	0	0.169	0.359	—

^*^Data of ages are presented as mean ± standard deviation, other data are presented as proportion.

^a^Fatal patients versus nonfatal severe patients, ^b^fatal patients versus mild patients, ^c^nonfatal severe patients versus mild patients.

Abbreviations: MODS, multiple organ dysfunction syndrome; DIC, diffuse intravascular coagulation.

As summarized in Table 2, leukopenia, especially neutropenia, thrombocytopenia and hypoeosinophilia, and increased levels of lactate dehydrogenase (LDH), creatine kinase (CK) and CK-MB were found in all patients. At the first week of disease, patients in fatal group showed significantly higher levels of viral load, CK, BUN and Cr, as well as lower platelet counts (PLT) and lower level of sodium ion (Na). Throughout the disease, the average viral load in fatal patients was 2–3 times higher than that in nonfatal patients (*P* = 0.000).

**Table 2:** Comparisons of laboratory characteristics between fatal and nonfatal patients from three to twenty days post the onset of severe fever with thrombocytopenia syndrome.

Index*	3–7 days		8–12 days		13–20 days	
	Fatal patients (N = 11)	Nonfatal patients (N = 22)	Fatal patients (N = 9)	Nonfatal patients (N = 22)	Fatal patients (N = 5)	Nonfatal patients (N = 20)
Viral load (log^10^)	7 (6–8)^a^	4(4–5)	6 (5.7–7.7)^a^	3 (2–3)	5 (4.7–7.7)^a^	2 (2–3)
PLT count (x10^9^/L)	33 (25–33)^b^	51(43.5–66)	29 (19–59)^b^	55 (36–68)	41 (23.5–61)^a^	156.5 (84–193)
WBC count (x10^9^/L)	2 (1.5–5.5)	2.1(1.76–3.9)	4.4 (3.5–12.3)	3.7 (2.6–6)	11.4 (6.2–16.6)^b^	4.6 (3.4–6.8)
EOS count (x10^9^/L)	0 (0–0.01)	0 (0–0.1)	0 (0–0.005)	0 (0–0.02)	0.01 (0.005–0.06)	0.02 (0.01–0.06)
HEM (%)	30.1 ± 6.4^b^	36.5 ± 4.7	33.4 ± 5.4	36.1 ± 4.4	27.6 ± 11.2	33.6 ± 5
LDH (U/L)	1879 (1078–2315)	1075 (633–1850)	1775 (1324–2770)^b^	879 (645–1563)	1311 (812–2517)^b^	335 (290–503)
CK (U/L)	648 (276–1592)^b^	309 (112–478)	1494 (401–3373)^b^	238.5 (155–649)	1283 (569–2170)^a^	68 (43–112.5)
CKMB (U/L)	54 (31–87)	17.5 (10.5–23.5)	63 (53–83.5)^b^	18.5 (13.8–28)	84 (28.5–110.5)^a^	11 (7–14.8)
ALT (U/L)	111 (73–190)	82.5 (48–189)	140 (70–259.5)	84 (51.5–165.5)	72 (42–118.5)	73.5 (52.3–119)
AST (U/L)	349 (100–572)	143.5 (103–512)	374 (89.5–647)	193 (83.3–482.8)	190 (95.5–424.5)^b^	68 (45.8–96)
BUN (mmol/L)	7 (6.3–10.7)^b^	5.8 (4.2–6.6)	7.5 (4.2–12.9)	4.7 (3.7–6.6)	13.9 (8.6–34.8)^a^	4.4 (3–5.2)
Cr (mmol/L)	97 (88–133)^b^	74.5 (60.3–88)	97 (53.4–148.5)	72.5 (60.5–83.3)	103 (74.5–118.5)^b^	50.5 (43.5–71)
Amylase (U/L)	158 (79–269)	135 (82.8–359)	229 (152–299)	137 (85–386)	357 (180–608.5)	125.5 (65–253)
Lipase (U/L)	608 (101–1169)	407 (129–1151)	512 (256–1277)	436.5 (118–861)	902 (306–2169)^b^	168.5 (73–572)
Na (mmol/L)	127.9 ± 4.9^b^	133.9 ± 4.8	127.7 ± 5.1^b^	133.9 ± 4.8	129.6 ± 4.1	135.1 ± 5.1
Proteinuria, N (%)	100	86.4	100	81.8	100^b^	30
Hematuria, N (%)	90.9	63.6	88.9	50	80^b^	5

^*^Data of HEM and Na are presented as mean ± standard deviation, data of Proteinuria and Hematuria are presented as proportion, other data are presented as median (interquartile range).

^a^ *p* < 0.001, ^b^ *p* < 0.05 com*p*ared with nonfatal patients.

Abbreviations: PLT, platelet; WBC, white blood cell; EOS, eosinophil; HEM, hematocrit; LDH, lactate dehydrogenase; CK, creatine kinase; ALT, alanine aminotransferase; AST, aspartate aminotransferase; BUN, blood urea nitrogen; Cr, creatinine; U/L, units/liter.

### Cytokines

**Figure 1:**
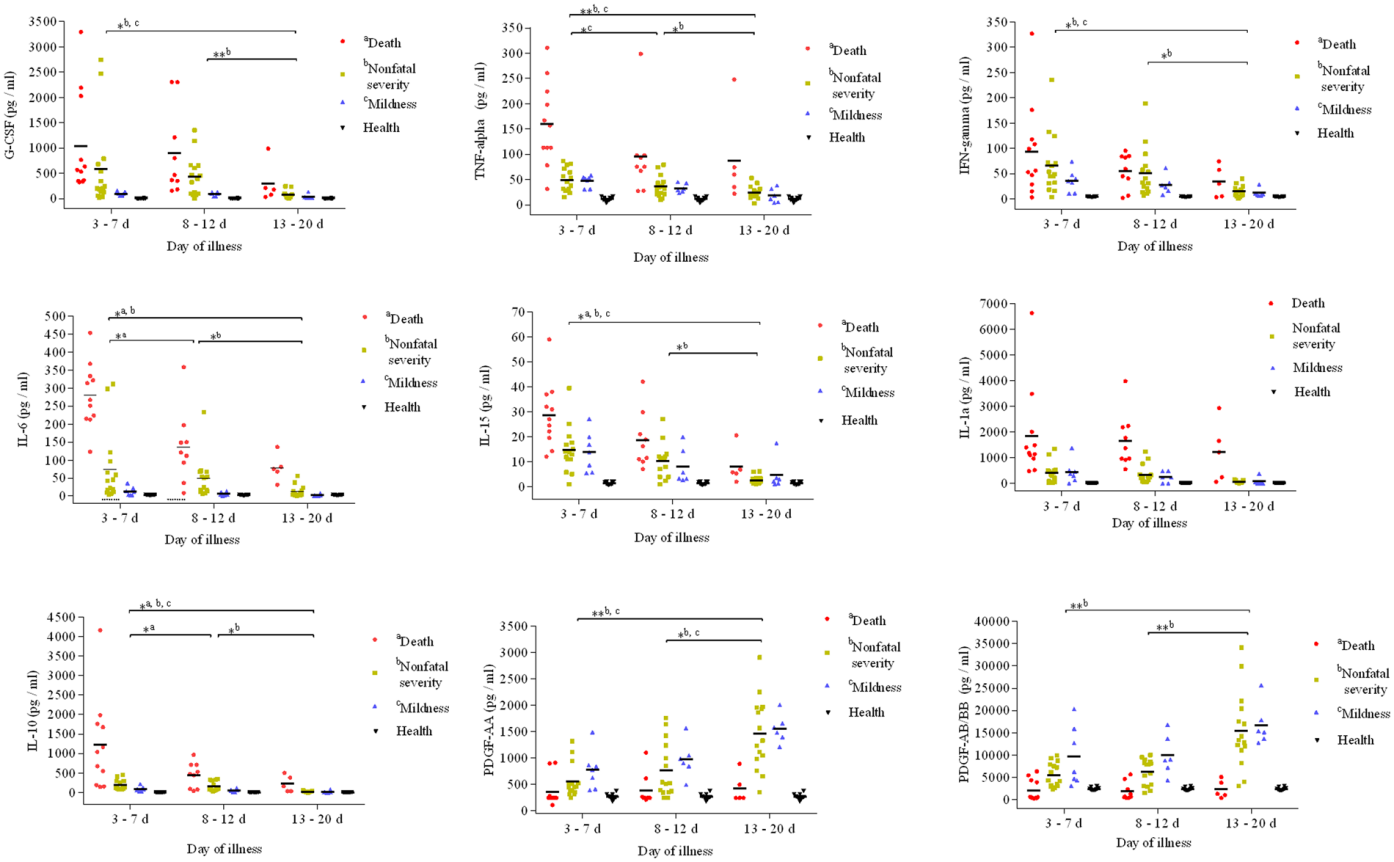
Dynamic changes of inflammatory cytokines were compared among fatal, nonfatal severe and mild patients throughout the course of severe fever with thrombocytopenia syndrome. Mild patients (*n* = 7): 3–7 d (*n* = 7), 8–12 d (*n* = 6), 13–20 d (*n* = 6); Nonfatal severe patients (*n* = 15): 3–7 d (*n* = 15), 8–12 d (*n* = 15), 13–20 d (*n* = 15); Fatal patients (*n* = 11): 3–7 d (*n* = 11), 8–12 d (*n* = 9), 13–20 d (*n* = 6); Healthy controls (*n* = 10). ^a^Comparison in death group, ^b^Comparison in nonfatal severity group, ^c^Comparison in mildness group. **P < 0.005, *P < 0.05. Abbreviations: IL-6, interleukin-6; IL-15, interleukin-15; TNF-α, tumour necrosis factor-alpha; G-CSF, granulocyte colony-stimulating factor; IFN-γ, interferon-gamma; IL-1α, interleukin-1α; IL-10, interleukin-10; PDGF-AA, platelet-derived growth factor-AA; PDGF-AB/BB, platelet-derived growth factor-AB/BB.

Among the 26 cytokines measured, the concentrations of IL-1RA, IL-6, IL-15, IL-10, TNF-α, IFN-γ and G-CSF were significantly elevated in SFTS patients compared to healthy controls at the first week of disease (Supplementary Table). As shown in Fig. 1, fatal patients exhibited a more robust production of cytokines than nonfatal patients. The levels of the 7 cytokines listed above in fatal patients were significantly higher than those in severe patients who survived. Additionally, no significant differences in these 7 cytokines were observed between nonfatal severe patients and mild patients.

**Figure 2:**
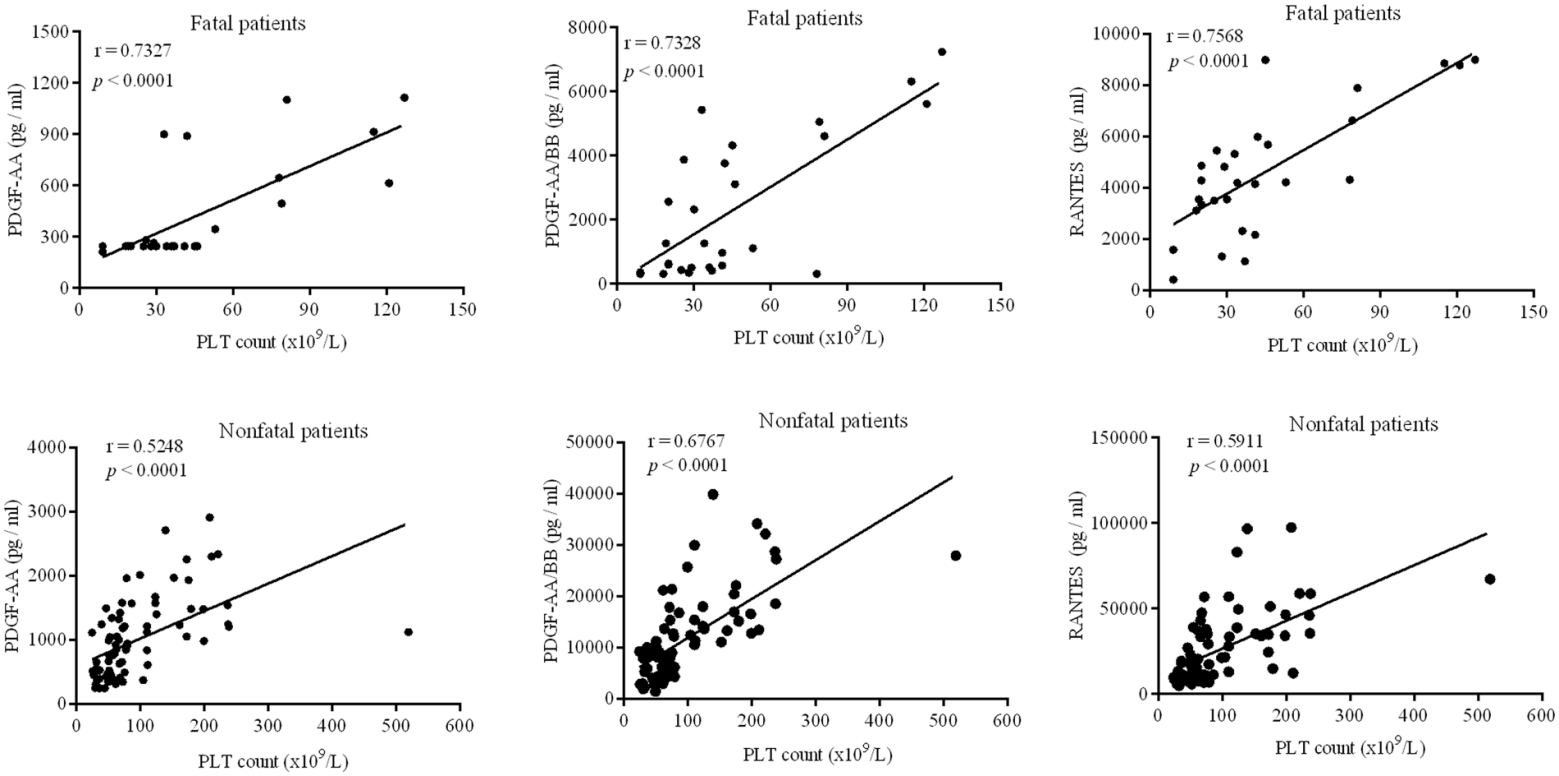
Correlation between PDGA level and platelet count as well as RANTES level and platelet count in patients of severe fever with thrombocytopenia syndrome. Logistic regression analysis was used to show the correlations between the PDGA level and platelet count as well as the RANTES level and platelet count. The best-fit lines are shown on each graph. On the graphs, r and *P* indicate the correlation coefficient and the *P*-value of significance, respectively. Abbreviations: PLT, platelet; PDGF, platelet-derived growth factor; RANTES, regulated on activation and normally T-cell expressed.

During the disease course in fatal patients, the 7 cytokines were at the highest levels on days 3–7. These levels did not decrease noticeably from days 3–7 to days 8–12, except for IL-6 and IL-10 (Fig. 1); only IL-6, IL-10 and IL-15 showed a significant decreasing trend from days 3–7 to days 13–20. The change trend of these 7 cytokines levels in nonfatal severe patients were similar as that in fatal patients throughout the disease. For mild patients, the levels of these cytokines were elevated slightly and were similar between days 3–7 and days 8–12, they reverted to their physiological ranges at the final stage of disease. Unlike other cytokines, PDGF were in low levels at the first week, throughout the disease progression, they then showed a significant increasing trend in nonfatal patients, but they keep low in fatal patients. The correlation analyses showed that significantly positive correlations existed between the PLT count and levels of PDGF in SFTS patients (Fig. 2).

### Chemokines

**Figure 3:**
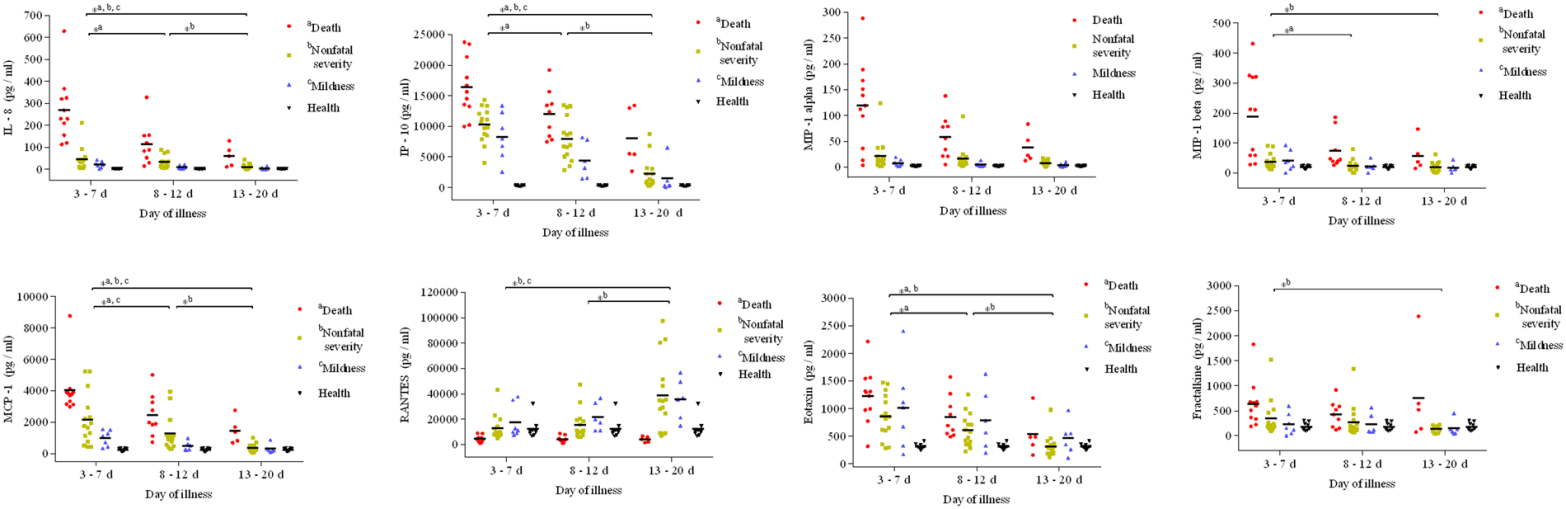
Dynamic changes of inflammatory chemokines were compared among fatal, nonfatal severe and mild patients throughout the course of severe fever with thrombocytopenia syndrome. Mild patients (*n* = 7): 3–7 d (*n* = 7), 8–12 d (*n* = 6), 13–20 d (*n* = 6); Nonfatal severe patients (*n* = 15): 3–7 d (*n* = 15), 8–12 d (*n* = 15), 13–20 d (*n* = 15); Fatal patients (*n* = 11): 3–7 d (*n* = 11), 8–12 d (*n* = 9), 13–20 d (*n* = 6); Healthy controls (*n* = 10). ^a^Comparison in death group, ^b^Comparison in nonfatal severity group, ^c^Comparison in mildness group. **P < 0.005, *P < 0.05. Abbreviations: IL-8, interleukin-8; IP-10, IFN-γ-inducible protein; MIP-1α, macrophage inflammatory protein-1a; MIP-1β, macrophage inflammatory protein-1β; MCP-1, monocyte chemotactic protein-1; RANTES, regulated on activation and normally T-cell expressed.

Among the 10 chemokines measured, the concentrations of eotaxin, IL-8, IP-10, MCP-1, MIP-1α, MIP-1β and fractalkine were significantly higher in patients compared to healthy controls (Supplementary Table) at the early stage of the illness. Compared with nonfatal patients throughout the disease course, IL-8, IP-10, MCP-1, and MIP-1α were significantly higher in fatal patients (Fig. 3). No significant differences in eotaxin, IL-8, IP-10, MCP-1, MIP-1α, MIP-1β and fractalkine were observed between nonfatal severe and mild patients during the progression of the disease.

In fatal patients, the levels of eotaxin, IL-8, IP-10, MCP-1 and MIP-1β decreased evidently from days 3–7 to days 8–12 (Fig. 3). RANTES decreased at the early stage and remained at a lower level throughout the disease in fatal patients. Significantly positive correlations existed between the PLT count and the level of RANTES in SFTS patients (Fig. 2). Nonetheless, the levels of IL-8, IP-10, MCP-1, MIP-1β, MIP-1α and RANTES did not return to their physiological ranges in fatal patients at the final stage of the disease except for eotaxin and fractalkine.

In nonfatal severe patients, the levels of eotaxin, IL-8, IP-10, MCP-1, MIP-1α, MIP-1β and fractalkine decreased gradually as patients recovered, whereas RANTES showed an increasing trend. The levels of eotaxin, IL-8, IP-10 and MCP-1 displayed a significant decreasing trend from days 8–12 to days 13–20. Significant differences in eotaxin, IL-8, IP-10, MCP-1, MIP-1β and RANTES were noted between days 3–7 and days 13–20 of the illness. In mild patients, only the levels of eotaxin, IL-8, IP-10, MCP-1 and MIP-1β were obviously elevated, and all of the chemokines reverted to normal levels when patients recovered. RANTES also displayed an upward tendency in mild patients.

## Discussion

In this study, the survived severe patients returned to convalescent stage at the third week. Renal damage at the early stage of the disease, hyponatraemia, consciousness disorder, and higher levels of CK and CK-MB were significantly associated with the aggravation of SFTS patients. Fatal patients were more significantly prone to incidence of hemorrhage, pulmonary infection, MODS and DIC. Vomiting and diarrhoea, which occurred more frequently in nonfatal patients, may indicate a better prognosis in SFTS.

Thus far, there are no specific treatments or preventive measures for SFTS, so study of its pathogenesis may illuminate appropriate treatment. As we know, the immune response plays an important role in the prognosis of infectious diseases, especially viral infections^10–12^. An appropriate inflammatory response is protective and essential for resistance to infection, dysregulated response can be detrimental, as excessive production of inflammatory mediators can cause vascular permeability, self-destruction of tissues and organ dysfunction^19^. Elevated inflammatory mediators have been reported to be associated with the severity of viral infections, such as IL-1β, IL-6, MCP-1, MIP-1α, MIP-1β, IL-2, IL-10, IFN-γ, TNF-α, IFN-α, IP-10, RANTES, IL-8 and IL-1RA in Ebola haemorrhagic fever^11,20–22^, IL-RA, TNF-α, IL-6, IL-1α, IFN-γ, IL-10, IL-8, IP-10 and MCP-1 in Dengue disease^23,24^, IL-6 and TNF-α in Crimean-Congo haemorrhagic fever^25^. IL-2, IL-6, IL-8, TGF-β1 and TNF-α in hantavirus infection^26^.

In this study, inflammatory mediators including IL-1RA, IL-6, IL-15, IL-10, TNF-α, IFN-γ, G-CSF, eotaxin, IL-8, IP-10, MCP-1, MIP-1α, MIP-1β and fractalkine were found to be elevated in SFTS patients during the first week of the disease. The inflammatory mediators retained high levels in fatal patients, while they recovered to normal levels gradually in nonfatal patients, especially decreased sharply in mild patients. Our results showed the occurrence of inflammatory storm were associated with the severity of disease in SFTS patients.

IFN-γ can inhibit viral replication, but aberrant expression is not conducive to disease recovery, the levels of IFN-γ were reported to be significantly lower in SFTS patients than in healthy individuals in a report by Deng^15^, while IFN-γ was reported to be elevated in the fatal cases during the acute phase of disease in other reports^13,14^. In this research, IFN-γ was found to be significantly elevated in SFTS patients, and this level decreased more rapidly in nonfatal patients than in fatal patients. Our results showed that TNF-α was elevated in SFTS patients and was significantly higher in fatal cases throughout the course of the disease, they significantly decreased on the second week in mild patients, while they decreased sharply on the third week in nonfatal severe patients. IL-6, another important pro-inflammatory cytokine which can induce B cells to differentiate into plasma cells, was significantly elevated in severe SFTS patients, it decreased gradually throughout the disease and recovered at the third week in nonfatal severe patients. IL-15 level was decreased significantly through the disease progression, it reverted to a near-normal level when patients recovered at the third week.

IL-1RA and IL-10, important anti-inflammatory cytokines, were significantly elevated in SFTS patients, especially in fatal patients. Throughout the disease, IL-10 showed significantly obvious downward trend, while IL-1RA showed a steadily descending trend. A compensatory production of anti-inflammatory mediators often occurs after the excessive production of inflammatory mediators. If inflammatory and anti-inflammatory mediators are significantly elevated and lose homeostasis, anti-inflammation response syndrome (CARS) or “immune paralysis” syndrome may result; as a consequence, patients will have increased susceptibility to secondary or opportunistic infections^27^. In this study, the peak levels of pro-inflammatory and anti-inflammatory cytokines were found to coexist during the first week in fatal patients, which may account for the higher incidences of MODS and pulmonary infection in fatal patients.

The significant role of IP-10 and MCP-1 in virus-infected disease progression has been reported^28^. In this study, although the levels of IP-10, IL-8 and MCP-1 declined significantly, they remained at high levels when death occurred in fatal patients. Elevated levels of MIP-1α and MIP-1β have been reported in fatal but not nonfatal SFTS patients^13,14^; however, our results showed MIP-1α and MIP-1β were significantly elevated not only in fatal patients but also in nonfatal severe patients. Although declined, these levels were still noticeably higher in fatal patients throughout the disease’s progression.

RANTES and PDGF are released by activated platelets^29^. Levels of RANTES were reported to be higher in SFTS patients than in healthy individuals by Deng^15^, while it was also reported to be significantly elevated in the fatal SFTS patients^13^. In our research, the concentrations of RANTES and PDGF were remarkably positively correlated with the PLT count in SFTS patients. The levels of RANTES and PDGF in fatal patients were significantly lower than nonfatal patients with the disease’s progression, suggesting that low levels of RANTES and PDGF are associated with an increased risk of death in SFTS patients.

Previous studies have reported that viral load is correlated with a strong immune response as well as the severity of disease following viral infection^13,14,30^. In this study, continuous detection revealed that the SFTSV load still remained high in fatal SFTS patients when death occurred.

Limitations of our study include the relatively small number of patients studied. However, this is a rigorous prospective study which took 2 years in a representative hospital for the treatment of SFTS, 37 inflammatory mediators were detected dynamically in SFTS patients as the disease progressed. Previous study have reported that type I interferon response would contribute to viral infection^16^, and this would need to be researched in our future study.

In summary, we firstly reported that the levels of IL-15 and eotaxin were elevated in SFTS patients. In total, abnormal production of 17 inflammatory mediators was observed in SFTS patients, they were associated with disease pathogenesis. In particular, sustained high levels of TNF-α, IL-6, IL-1RA, IL-8, IP-10, MCP-1, MIP-1α, MIP-1β, and low production of RANTES and PDGF were most associated with the severity of disease. Due to the lack of specific antiviral drugs against SFTSV, treatments such as blood purification, glucocorticoids and antagonists to block the cytokine storm might relieve the disease severity and decrease SFTS mortality.

## Electronic supplementary material

supplementary

supplementary dataset
