# Supplementary material for: The Regulation of Seventeen Inflammatory Mediators are Associated with Patient Outcomes in Severe Fever with Thrombocytopenia Syndrome
Source: Sci Rep. 2018 Jan 9;8:159. doi: 10.1038/s41598-017-18616-z (PMC5760584; doi:10.1038/s41598-017-18616-z)
Supplement: Supplementary file 1 — supplementary [file 41598_2017_18616_MOESM1_ESM.pdf]

The Regulation of Seventeen Inflammatory Mediators are Associated with Patient Outcomes in Severe Fever with Thrombocytopenia Syndrome

Running title: Inflammatory Reaction in SFTS

Li-Fen Hu[1], Ting Wu[1], Bo Wang[1], Yuan-Yuan Wei[1], Qin-Xiang Kong[2], Ying Ye[1], Hua-Fa Yin[1], and Jia-Bin Li[1, 2]*

[1]Department of Infectious Diseases, the First Affiliated Hospital of Anhui Medical University, Hefei, Anhui, China;

[2]Department of Infectious Diseases, the Chaohu Affiliated Hospital of Anhui Medical University, Hefei, Anhui, China.

Li-Fen Hu and Ting Wu contributed equally to this work.

***Corresponding author:** Jiabin Li

Department of Infectious Diseases, the First Affiliated Hospital and the Chaohu Affiliated Hospital of Anhui Medical University, Jixi road 218, Hefei, Anhui, China

Tel: +86-551-62922713, Fax: +86-551-62922281, E-mail: lijiabin948@vip.sohu.com

Table. Comparisons of inflammatory mediators between patients of severe fever with thrombocytopenia syndrome and controls of health.

Index	3 -7 days			8 -12 days			13 -20 days		
	p [a]	p[b]	p[c]	p[a]	p[b]	p[c]	p[a]	p[b]	p[c]
IL-1ra	0.004	0.004	0.006	0.000	0.009	0.003	0.004	0.002	0.131
IL-6	< 0.0001	0.031	0.014	0.001	0.015	0.047	< 0.0001	0.067	0.784
IL-10	0.004	0.000	0.000	0.000	0.000	0.001	0.005	0.000	0.078
IL-15	< 0.0001	0.000	0.000	0.000	0.000	0.009	0.009	0.031	0.103
G-CSF	0.001	0.012	< 0.0001	0.002	0.003	< 0.0001	0.029	0.007	0.029
IFNgamma	0.007	0.003	0.000	0.000	0.007	0.001	0.008	0.006	0.012
TNFalpha	< 0.0001	< 0.0001	< 0.0001	0.004	0.001	< 0.0001	0.017	0.004	0.161
PDGF-AA	0.730	0.011	0.001	0.348	0.005	< 0.0001	0.150	< 0.0001	< 0.0001
PDGF-AB/BB	0.305	0.047	0.005	0.838	0.013	0.000	0.560	0.001	< 0.0001
Eotaxin	< 0.0001	0.001	0.012	< 0.0001	0.005	0.384	0.089	0.940	0.125
IL-8	< 0.0001	0.010	0.000	0.002	0.002	0.012	0.002	0.021	0.135
IP-10	< 0.0001	< 0.0001	< 0.0001	< 0.0001	< 0.0001	0.000	0.000	0.540	0.152
MCP-1	< 0.0001	0.001	0.000	< 0.0001	0.009	0.034	0.000	0.286	0.516
MIP-1alpha	0.000	0.058	0.013	0.000	0.063	0.061	0.015	0.002	0.086
MIP-1beta	0.002	0.040	0.071	0.012	0.577	0.725	0.037	0.814	0.579

Fractalkine	0.006	0.291	0.529	0.012	0.403	0.580	0.069	0.092	0.560
RANTES	0.003	0.411	0.318	0.014	0.249	0.061	0.048	0.007	0.001

[a] Death versus health, [b] Nonfatal severity versus health, [c] Mildness versus health.

Abbreviations: IL-1α, interleukin -1α; IL-6, interleukin -6; IL-10, interleukin-10; IL-15, interleukin-15; G-CSF, granulocyte colony-stimulating factor; IFN-gamma, interferon-gamma; TNF-alpha, tumor necrosis factor-alpha; PDGF-AA, platelet-derived growth factor-AA; PDGF-AB/BB, platelet-derived growth factor- AB/BB; IL-8, interleukin-8; IP-10, IFN-γ-inducible protein; MIP-1α, macrophage inflammatory protein-1a; MIP-1β, macrophage inflammatory protein-1β; MCP-1, monocyte chemotactic protein-1; RANTES, regulated on activation and normally T-cell expressed.
